# Hospital readmissions in Internal Medicine Specialty: Frequency, associated factors and outcomes

**DOI:** 10.12669/pjms.37.7.3575

**Published:** 2021

**Authors:** Samar Fatima, Sara Shamim, Simra Raffat, Muhammad Tariq

**Affiliations:** 1 Dr. Samar Fatima, FCPS. Department of Medicine, Aga Khan University Hospital, Karachi, Pakistan; 2 Dr. Sara Shamim, MBBS, MBA. Department of Medicine, Aga Khan University Hospital, Karachi, Pakistan; 3 Dr. Simra Raffat, MBBS. Department of Medicine, Aga Khan University Hospital, Karachi, Pakistan; 4 Dr. Muhammad Tariq, MRCP, FACP, FRCP, FRCP, MHPE. Department of Medicine, Aga Khan University Hospital, Karachi, Pakistan

**Keywords:** Hospital readmission, Index admission, Discharge

## Abstract

**Objectives:**

Hospital readmission has become a focus of national attention as a potential indicator of healthcare quality and has a significant financial impact on healthcare system. Limited data is available regarding readmissions to Internal Medicine specialty from our sub-continent. It is, therefore, essential to determine the frequency and factors leading to readmissions, in order to avoid preventable readmissions and improve quality of healthcare provision.

**Methods:**

This retrospective study reviewed adult discharges from Internal Medicine specialty between October 2018 and February 2019 at Aga Khan University Hospital. Out of 1,835 discharges, 491 were randomly selected after excluding expiries. The frequency, factors and outcomes of readmission were noted. The studied outcomes included length of stay and in-hospital mortality.

**Results:**

Out of 491 patients, 15.3% were readmitted within 30-days of their discharge. Most of the readmitted patients were females (56%) and elderly with a mean age 67.1±14.9 years. Majority of the patient who got readmitted had multi-morbidities (68%) and were of functional Class-II (39%).The mean length of stay for index and readmission was between 4-7days. Eighty-percent readmissions were discharged as planned, 13% on request and seven-percent left against medical advice in their index admission. The most common causes of readmission were persistence of symptoms (43%) and nosocomial infection (29%). Avoidable causes included hospital-associated pneumonia, urinary tract infections and septic shock. Mortality in readmitted patients was 12%.

**Conclusions:**

The causes of readmission is multi-factorial, including advanced age, multi-morbidities, persistence of symptoms and nosocomial infections. Early follow-ups should be advised to prevent avoidable readmissions.

## INTRODUCTION

Hospital readmissions is considered as a major focus of quality care as it puts an unnecessary physical, social, and emotional burden on patients, incurring additional costs of several billion dollars annually.^1^-^4^ It is hypothesized that patients who are readmitted within few days after discharge might have experienced suboptimal treatment and care in their previous admission.^1^ Therefore, these factors should be identified, for better care provision.

Overall readmission rate reported in patients admitted to Internal Medicine services of six academic medical centers(USA) was 17.5%^5^ and 22.8%.^6^ The cause of readmissions could be regardless of primary diagnosis,^7^ it could be potentially avoidable and preventable with appropriate medical care.^8^,^9^

In developed countries, healthcare expenditure is typically payable through insurance. However, in a low socioeconomic country like Pakistan, this cost is usually paid by single income-producer of family, who bears financial burden of his own as well as his family members. Frequent hospitalizations put him through further financial hardships.

Although local studies has been done to identify specialty-specific readmission rates,^10^,^11^ and concerning to sepsis^12^ and heart failure patients^13^ this data is lacking for Internal Medicine specialty and this may be due to lack of infrastructure to maintain patient’s database.

Internal Medicine patients are more complicated as compared to other specialty based on their older age, multi-morbidities, and functional status. However, there is limited data for patients admitted to Internal Medicine specialty in our sub-continent. The differences in ethnicity, socioeconomic background, and hospital system differences may lead to variation in readmission rates, and factors.^14^,^15^ Therefore, this study was conducted to determine frequency, associated factors and outcomes of readmissions in Internal Medicine patients within 30-days of index admission to avoid preventable readmissions and improve healthcare quality.

## METHODS

This retrospective study was carried out at Aga Khan University Hospital. It is one of Pakistan’s largest tertiary-care hospital and caters to a diverse group of patients from different socioeconomic backgrounds.

All patients aged above 18-years admitted electively from clinics or through emergency department (ED) under care of Internal Medicine specialty from October 2018 to February 2019 were identified using ICD coding. Out of 1835 cases, every 3^rd^ patient was selected for review, representing a sample size of 491. Patients who returned after 30-days of their discharge or those expired in their index admission along with those admitted to the subspecialties were excluded.

Index admission referred to initial admission and defined as first hospitalization out of multiple during the study period. However, readmission was defined as when a patient, after discharge from hospital, was admitted again within 30-days of discharge from initial admission.

Data included detailed patient-level information such as patient’s demographics including age and gender followed by co-morbid conditions, functional status, admission mode (Emergency or elective), discharge mode (definitive discharge, discharge on request or LAMA). Length of stay in index admission and readmission, follow-up in the clinic after the discharge (from index admission) and patient expired after readmission were also reviewed and documented in structured performa from their medical and electronic records.

The diagnosis at initial discharge and on readmission was also noted. The causes of readmission were further inquired and categorized as: persistence of symptoms, nosocomial infection, development of disease in the new system, complication of treatment, or missed diagnosis (according to their best judgment as to the relationship from the initial to the subsequent admission).

Persistence of symptoms was defined as the continuation of same symptoms of illness as of index admission. Nosocomial infection, also known as hospital-acquired infection, was defined as an infection that was acquired in the hospital, however manifested after discharge and lead to readmission (example patient was admitted in index admission with diagnosis of congestive heart failure and was readmitted with diagnosis of Hospital acquired Pneumonia). The most common causes of nosocomial infection are direct contact, droplet transmission, airborne transmission and common vehicle transmission. The development of disease in the new system was defined as disease not related to the initial diagnosis, and neither due to any complications acquired in the hospital. The complication of treatment was referred to as any complication related to the care provided in index admission while missed diagnosis referred to as an incorrect diagnosis about the cause of disease during the index admission.

There is frequent need of rehabilitation in patients discharged from the hospital, especially in elderly, due to complexity of their health problems, comorbidities, polypharmacy, and limitations in performing daily activities. In this manuscript we defined rehabilitation as those requiring nursing care, physiotherapy, bed sore care, suctioning, and nebulization.

This study was approved by Ethical Review Committee (Approval # 2019-0893-2214) of the hospital, dated January 15^th^, 2019.

### Statistical Analysis

Data was entered and analyzed using SPSS version24. Frequencies were calculated for categorical variables. Means and standard deviations were calculated for quantitative variable.Comparative analysis was done using an independent sample t-test or chi-square test where appropriate. All p-values were based on two-sided tests and a significant p-value of <0.05.

## RESULTS

Total of 491 admissions were reviewed, out of which 450(91.6%) patients were admitted through ED.

### Frequency, associated factors and outcomes of readmitted patients

Overall, 75(15.3%) patients got readmitted within 30-days of their discharge from index admission.

Most readmitted patients were female (56%) and elderly with a mean age 67.1±14.9 years [P-value<0.001], among them 24% were above 80 years of age (Table I and II). Most common comorbidities were HTN (73%), DM (56%), CKD (28%), and IHD (21%). It was observed that patients with diabetes and hypertension were more likely to get readmitted with significant p-values. Most of the patient (68%) who got readmitted had more than two comorbidities and were of functional Class-II (39%).

**Table I:** Characteristics of study population (n=491).

	*Total*	*Non-readmitted N=416 (84.7%)*	*Readmissions N=75(15.3%)*	*P-values*
Age, in years	58.0±19.2	56.4±19.5	67.1±14.9	<0.001
***Gender***				
Male	232(57.3)	199(47.8)	33(44)	0.54
Female	259(52.7)	217(52.2)	42(56.0)	
***Diabetes***				
Yes	221(45.0)	179(43.0)	42(56.0)	0.03
No	270(55.0)	237(57.0)	33(44.0)	
***Hypertension***				
Yes	268(54.6)	213(51.2)	55(73.3)	<0.001
No	223(45.4)	203(48.8)	20(26.7)	
***Ischemic Heart Disease***				
Yes	104(21.2)	88(21.2)	16(21.3)	0.97
No	387(78.8)	328(78.8)	59(78.7)	
***Atrial Fibrillation***				
Yes	19(3.9)	13(3.1)	6(8.0)	0.04
No	472(96.1)	403(96.9)	69(92.0)	
***Chronic Kidney Disease***				
Yes	74(15.1)	53(12.7)	21(28.0)	0.001
No	417(84.9)	363(87.3)	54(72.0)	
***Cerebrovascular Accident***				
Yes	33(6.7)	25(6.0)	8(10.7)	0.13
No	458(93.3)	391(94.0)	67(89.3)	
***Asthma***				
Yes	21(4.3)	14(3.4)	7(9.3)	0.01
No	470(95.7)	402(96.6)	68(90.7)	
***Chronic Obstructive Pulmonary Disease***				
Yes	23(4.7)	13(3.1)	10(13.3)	<0.001
No	468(95.3)	403(96.9)	65(86.7)	
***Tuberculosis***				
Yes	23(4.7)	12(2.9)	11(14.7)	<0.001
No	468(95.3)	404(97.1)	64(85.3)	
***Malignancy***				
Yes	34(6.9)	23(5.5)	11(14.7)	0.004
No	457(93.1)	393(94.5)	64(85.3)	
***Chronic Liver Disease***				
Yes	15(3.1)	12(2.9)	3(4.0)	0.60
No	476(96.9)	404(97.1)	72(96.0)	

**Table II:** Associated factors and outcomes of readmitted patients.

*Characteristics*	*Readmissions N=75(%)*
Patients >80 years	18(24)
***Comorbid conditions***	
≤2	24(32)
>2	51(68)
***Functional Class***	
I-II	52(69.4)
III-IV	23(30.6)
***Admission Mode***	
Emergency	70(93)
Elective	5(7)
***Discharge Mode***	
Definitive discharge	60(80)
Discharge on request	10(13)
LAMA	5(7)
***Length of stay (index admission)***	
1-3days	13(17)
4-7days*	44(59)
8+	18(24)
***Length of stay (readmission)***	
1-3days	15(20)
4-7days*	48(64)
8+	12(16)
Patients requiring rehabilitation	24(32)
Patients discharged on IV-medications	30(40)
Patients discharged on urinary catheter	8(11)
***Readmission status***	
Readmitted within 7days	28(37.3)
Readmitted in 8-30days	47(62.7)
***Follow-up in clinic after discharge***	
Within 2weeks	13(17)
Missed follow-up	62(83)
Mortality after readmission	9(12)

* Mean(SD) 5(4-7) days.

All readmissions were analyzed based on their admission and discharge mode. It was found that majority (93%) were admitted through ED and most of the readmissions (80%) had definitive discharge in their index admission. The majority (59-64%) of the readmitted patients had a duration of hospital stay of 4-7days in both their index admission and readmission. During the index admission, 32% of readmitted patients required rehabilitation, 40% patients were discharged on IV medications, and 11% were discharged on urinary catheters.

Follow-ups were missed in 83% patients (as per discharge recommendation). However, it was observed that 37.3% got readmitted within 7-days of discharge before their scheduled follow-up. Mortality of readmitted patients was 12% (Table-II). The cause of mortality in readmitted patients were persistence of symptoms (n=5) and nosocomial infection (n=4).

### Patient diagnoses at index admission and readmission

Frequent diagnoses of readmitted patients at index admission included urinary tract infections, Pneumonia, Myocardial infarction, pulmonary edema, and acute exacerbation of COPD/Asthma. However, the most common diagnoses of readmission were persistence of previous symptoms (43%) and nosocomial infections (29%), followed by disease into a new system (24%) and complication of treatment (3%). While only one patient had missed diagnoses (Table-III).

**Table III:** Analysis of readmitting diagnosis.

	*Readmission N=75(%)*	*Within 7days N=63(%)*	*Within 30-days N=12(%)*
Persistent symptoms	32(43)	29(46)	3(25)
Nosocomial infection	22(29)	16(25)	6(50)
Disease in a New System	18(24)	17(27)	1(8)
Complication of treatment	2(3)	0(0)	2(17)
Missed Diagnosis	1(1)	1 (2)	0(0)

Most common avoidable diagnosis at readmission included nosocomial infections (Table-IV). On further stratification, it was observed that 60% of the patients who left against medical advice and 52% of the patients who missed their follow-ups, bounced back with persistence of previous symptoms.

**Table IV:** Avoidable diagnosis of readmission.

*Causes*	*Readmission=25(%)*
Hospital-Acquired Pneumonia	12(48)
Urinary Tract Infection	5(20)
Septic shock	5(20)
Infected bedsores	3(12)

## DISCUSSION

Hospital readmission has become a focus of national attention as a potential indicator of poor quality and healthcare waste. This observational study focused on frequency and readmission factors in a tertiary-care hospital in a low middle-income country, which is increasingly essential yet poorly studied.

The majority of the people in Pakistan belong to the middle-class, earning between two-thirds and double their median household income. Cost of readmission is usually self-paid and less likely from insurance companies. By identifying contributing risk factors leading to readmissions, during index admission, we can prevent an unexpected return of patients, which are associated with high morbidity and mortality.^6^,^16^-^18^

Our study’s overall readmission frequency is comparable with findings of three relevant studies in USA (14.7-17.5%). In these studies, only elderly population was selected to assess the readmission risk,^5^,^19^ however current study included all adult patients of Internal Medicine specialty irrespective of their age or payment modes. Although local studies have been done to identify specialty-specific readmission rates,^10^,^11^ this data is lacking for Internal Medicine specialty. In a local study done on sepsis patients, the readmission rate was found to be 42% within 180days,^12^ however our study recruited all adult Internal Medicine patients readmitted with 30 days after their discharge from initial admission.

Our study showed that readmissions were frequent in patients with older age, multi-morbidities and those requiring advanced care (patients requiring rehabilitation or IV-medications at home after discharge). Results are comparable with work done by Donzé et al., who finds that primary diagnosis is directly or indirectly related to patient’s specific comorbid condition.^8^ However, several other studies suggest that patient’s socio-demographic characteristics are associated with readmissions,^15^ but this factor is not considered a dominant cause of readmission in this study.^14^,^20^ Literature shows that impaired functional status is also associated with frequent hospitalizations.^6^,^21^ However, we find a noticeably smaller correlation with impaired functionality and readmission.

The study contributes to the existing work carried out by Koekkoek et al., who identified the factors of preventable readmission and found that they are influenced by prominently by process factors and patient factors.^9^ Complex comorbid conditions, advanced age, impaired functional status, progressive disease, and lack of rehabilitation services at home are few patient-related factors.^8^,^9^,^21^-^23^ The process factors include rapid discharge processes and hospital-acquired infections at index admission.^24^ Therefore, some healthcare systems are strategizing to build predictive models for earlier identification of patients at high-risk of readmission.^25^

This study found that most of the readmitted patients, presented with the persistence of the symptoms (as in their prior admission). Nosocomial infections were the second most common cause of readmissions, followed by developing the disease in a new system. It extends the findings of other studies done in developed countries that examined the causes of readmission that could be regardless of original admitting diagnosis.^7^,^13^

Like other studies,^26^ length of stay in readmitted patients at index admission and readmission in this study were prolonged compared to the hospital benchmark (Excellent performance target for length of stay is 3.8±2.8days whereas Acceptable performance target is 4.2±2.8days). This finding could have been due to advanced age, multi-morbidities, rehabilitation issues, and difficulties of recovery in readmitted patients. It could have predisposed them to an increased risk of nosocomial infections. In contrast, mortality rate in readmitted patients in this study is slightly lower than in comparative studies.^6^

Prevention of readmission and mortality after hospitalization is complicated. Our findings hold important implications for policymakers. We may reduce readmission frequency by concentrating on the at-risk group. Discharge process should focus not only on acute conditions responsible for index admission but also on patients’ underlying comorbid conditions that may increase risk of new, acute complications for patients. Before discharge, palliative care, home healthcare, and rehabilitation service should be taken into loop. Also, close follow-up within 4-5 days of discharge should be arranged to avoid readmission-risk. It can be implemented as a policy in hospital that can become active before discharge. Risk of nosocomial can be reduced by decreasing length of stay, proper hand hygiene and early removal of urinary catheters. The increased length of stay during next admission is associated with mortality rather than it leads to mortality.

### Limitations of the study

Few limitations include a single-centered study with comparatively small sample size and focused only on one specialty. Readmission frequency may have been underestimated as patients discharged in their index admission from this healthcare facility might have been readmitted to another healthcare facility. Due to improper documentation, we couldn’t figure-out the root cause of septic shock. Mortality of readmission is only predictive of in-hospital mortality, however, it doesn’t capture mortality if it occurred outside the hospital setting.

## CONCLUSION

This study discusses the incidence and readmission variables and methods for reducing readmissions frequency and avoiding preventable readmissions. According to our results, elderly, diabetic, hypertensive, and those with multi-morbidities are the patients at risk of getting readmitted. Patients admitted for more than 4-days in their index admission, those requiring rehabilitation and IV-medications on discharge are at higher readmission-risk. The persistence of symptoms followed by nosocomial infections are the most common causes of readmissions. The patients who request early discharge, LAMA and missed follow-ups are more likely to be readmitted.

### Authors’ Contribution:

**SF & SS:** Conceived, designed and did statistical analysis & manuscript writing and editing. Also responsible for accuracy and integrity of the work.

**SR:** Did data collection.

**MT:** Did review and final approval of manuscript.
